# Cytokine responses of CD4^+ ^T cells during a *Plasmodium chabaudi chabaudi *(ER) blood-stage infection in mice initiated by the natural route of infection

**DOI:** 10.1186/1475-2875-6-77

**Published:** 2007-06-07

**Authors:** Luis Fonseca, Elsa Seixas, Geoffrey Butcher, Jean Langhorne

**Affiliations:** 1 Division of Parasitology, National Institute for Medical Research, London NW7 1AA, UK; 2 Instituto Gulbenkian de Ciência, Portugal; 3 Faculty of Natural Sciences, Division of Cell and Molecular Biology, Imperial College, London SW7 2AZ, UK

## Abstract

**Background:**

Investigation of host responses to blood stages of Plasmodium spp, and the immunopathology associated with this phase of the life cycle are often performed on mice infected directly with infected red blood cells. Thus, the effects of mosquito bites and the pre-erythrocytic stages of the parasite, which would be present in natural infection, are ignored In this paper, *Plasmodium chabaudi chabaudi *infections of mice injected directly with infected red blood cells were compared with those of mice infected by the bites of infected mosquitoes, in order to determine whether the courses of primary infection and splenic CD4 T cell responses are similar.

**Methods:**

C57Bl/6 mice were injected with red blood cells infected with *P. chabaudi *(ER) or infected via the bite of *Anopheles stephensi *mosquitoes. Parasitaemia were monitored by Giemsa-stained thin blood films. Total spleen cells, CD4+ T cells, and cytokine production (IFN-γ, IL-2, IL-4, IL-10) were analysed by flow cytometry. In some experiments, mice were subjected to bites of uninfected mosquitoes prior to infectious bites in order to determine whether mosquito bites *per se *could affect a subsequent *P. chabaudi *infection.

**Results:**

*P. chabaudi *(ER) infections initiated by mosquito bite were characterized by lower parasitaemia of shorter duration than those observed after direct blood challenge. However, splenomegaly was comparable suggesting that parasitaemia alone does not account for the increase in spleen size. Total numbers of CD4 T cells and those producing IFN-γ, IL-10 and IL-2 were reduced in comparison to direct blood challenge. By contrast, the reduction in IL-4 producing cells was less marked suggesting that there is a proportionally lower Th1-like response in mice infected via infectious mosquitoes. Strikingly, pre-exposure to bites of uninfected mosquitoes reduced the magnitude and duration of the subsequent mosquito-transmitted infection still further, but enhanced the response of CD4 T cells producing IFN-γ and IL-4.

**Conclusion:**

The data in this paper suggest that studying early host responses in blood stage malaria infections measured after direct blood challenge of mice may not completely reflect the natural situation, and more detailed investigations of blood-stage immunity after mosquito transmission in experimental models should be considered.

## Background

Non-lethal malaria infections in mice directly infected with blood stage parasites are characterised by parasitaemia sometimes exceeding 40% of infected erythrocytes and an acute inflammatory response [1]. Much of pathology at this time is thought to be a consequence of the production of pro-inflammatory cytokines [2,3]. These cytokines can be induced by direct interaction between the parasite and dendritic cells, monocytes and macrophages [4,5] resulting in NK, γδ and Th1 CD4^+ ^T cell activation and the further release of cytokines such as IFN-γ, TNF-α and LT [2,6]. However, it is not known whether these strong pro-inflammatory responses are, in part, a result of high initial parasitaemia that may not occur when the infection is initiated by the natural route of mosquito infection, and also whether the pre-existing sporozoite and pre-erythrocytic forms affect in any way the blood stage infection or the host's immune response to it.

Sporozoites migrate rapidly to the liver where they invade hepatocytes and initiate pre-erythrocytic schizont development. A blood stage infection begins approximately two days later, after rupture of the mature liver schizont, and release of merozoites, which then invade erythrocytes and establish the erythrocytic cycle. This exposure of the host to malarial antigens and parasite Pathogen-associated Molecular Patterns (PAMPs) [7], in an environment such as the liver, before the erythrocytic stage of the infection may well have an impact on the subsequent innate and acquired immune response to the blood stages. Although the liver is not a secondary lymphoid organ, it is likely to be a site where phagocytic cells, such as Kuppfer (cells (KC) and dendritic cells (DC), encounter and take up sporozoites. It does enlarge with multiple infections and is a site of phagocytosis of infected and uninfected red cells [8]. The liver environment is considered to be tolerogenic [9] and could, therefore, influence APC activation and presentation, and thus the nature and magnitude of the CD4^+ ^T cell response to those antigens seen later in the blood stages. The interactions of DC from the liver with malaria parasites have not been studied, but naïve KC are not activated by infectious sporozoites to produce IL-12p40 and antigen-presenting capacity is impaired [10]. Since CD4^+ ^T cells are important for the development of protective immunity and contribute to pathology during blood stage infection, it is important to know if pre-erythocytic stage infections affect this response.

A primary blood stage infection of a clone of *Plasmodium chabaudi*, (ER), following direct injection of infected red blood cells (pRBC) was compared with a blood stage infection after natural transmission via inoculation of sporozoites by *Anopheles stephensi *mosquitoes. Blood parasitaemia and cytokine production was measured in splenic CD4^+ ^T cells at early and later stages of a primary blood stage infection. Overall, the parasitaemia following sporozoite challenge were lower than those observed after direct blood inoculation. Despite this, there was an early IFN-γ response from CD4^+ ^T cells, albeit reduced in comparison with the response to direct blood challenge. Interestingly, uninfected mosquito bites given prior to exposure to infected mosquitoes resulted in a delayed and lower parasitaemia accompanied by an increased IFN-γ response from CD4^+ ^T cells.

## Materials and methods

### Mice and parasites

Female C57BL/6 mice, aged 6–8 weeks from Charles River (Margate, Kent, U.K.) or from the specific pathogen free breeding facilities at The National Institute for Medical Research (NIMR), London, were infected with *P. c. chabaudi *(ER) parasites, originally obtained from David Walliker (University of Edinburgh, UK). Parasitaemia were monitored by Giemsa-stained thin blood films.

### Maintenance of mosquitoes

*Anopheles stephensi *(strain SD500) female mosquitoes bred at the insectary of the Cell and Molecular Biology Division, Biology Department at Imperial College for Science Technology and Medicine (London,) maintained as described [11,12] were kindly provided by Jacqui Mendoza. Groups of 50 mosquitoes in paper cups were given a solution of 80 g/l D (-) fructose (Sigma, U.K.) and 0.5 g/l p-amino benzoic acid (PABA) (Sigma, U.K.) in H_2_O, placed inside a cooled incubator with the temperature maintained between 24–27°C, relative humidity higher than 50 % and a light cycle of 12 hours light/12 hours dark.

### Infection of mosquitoes

Mice (group size of 5) were injected *i.p*. with 10^5 ^parasitized erythrocytes. Six and fourteen days later, the presence of gametocytes in mice was determined through Giemsa-stained thin blood films. Mice were anaesthetized by ip injection of a mixture of Rompum (Bayer, Suffolk, UK) and Vetalar (Parke-Davis Veterinary, Gwent, UK) [13], and placed on the net covering the top of the paper cups, ventrally exposed to the mosquitoes. The feeding session lasted for 20 min and every 5 min mice were transferred to other paper cups, to ensure that all the cups of mosquitoes would be exposed to all the mice and vice-versa. At the end of the feeding session, after removal of those mosquitoes that did not feed, mosquitoes were returned to the incubator.

### Infection of mice by mosquito bite

To verify if the mosquitoes were infected, on day 8 and 10 after feeding mosquitoes on infected mice, the stomachs from 10 mosquitoes from several paper cups were dissected and examined under a microscope for the presence of oocysts. Fourteen days after feeding mosquitoes on infected mice, transmission of the parasites from those mosquitoes to uninfected mice was carried out, but only after confirming that sporozoites were present in the salivary glands of a sample of 10 mosquitoes by mosquito dissection. The feeding session proceeded as described above.

### Dissection of salivary glands and sporozoite injection

Mosquito salivary glands, isolated under a binocular magnifier were pooled and disrupted using a glass homogeniser (Merck, UK). The number of sporozoites was calculated using an Improved Neubauer chamber (Weber Scientific International Ltd, UK) and different numbers of sporozoites were then injected into the tail vein of mice.

### Preparation of cell suspensions for surface and intracellular staining

Single cell suspensions of spleens were added erythrocyte lysis buffer containing 0.16 M NH_4_Cl (Sigma, UK) and 0.17 M Tris (Sigma) pH 7.65, at a final cell concentration of approximately 10^7 ^cells/ml, and incubated for 10 min at room temperature. Cells were then centrifuged and the total number of cells obtained was counted using an Improved Neubauer chamber. the cells were then placed in 96 wells plates (NUNC, UK) at a concentration of 2 × 10^5 ^cells per well.

### Stimulation of cells

Cells were stimulated using Phorbol 12-myristate 13-acetate (PMA) and ionomycin (Calbiochem, UK) at final concentrations of 50 ng/ml and 500 ng/ml respectively and incubated for two hours at 37°C and 5 % CO_2_. Cells were then further incubated for two hours with Brefeldin A (Sigma) at a final concentration of 10 μg/ml

### Surface and intracellular staining of cells

Antibodies to CD4, IL-2, IL-4, IL-10, IFN-γ and appropriate isotype controls labeled with FITC or PE were obtained from BD Bioscience, UK. Surface and intracellular staining was carried out in the presence of normal rat IgG and anti-FcR block (BD Bioscience, UK) to prevent nonspecific and FcR-mediated binding of Ig as described previously [14]. Cells were acquired on a FacsCalibur, (BD Bioscience). 50,000 viable cells (gated on 90° and forward light scatter for viable lymphocytes) were acquired and analysed using Cell Quest software (BD Bioscience).

## Results

### Establishment of mosquito transmission of *P. chabaudi chabaudi *(ER)

**Figure 1:**
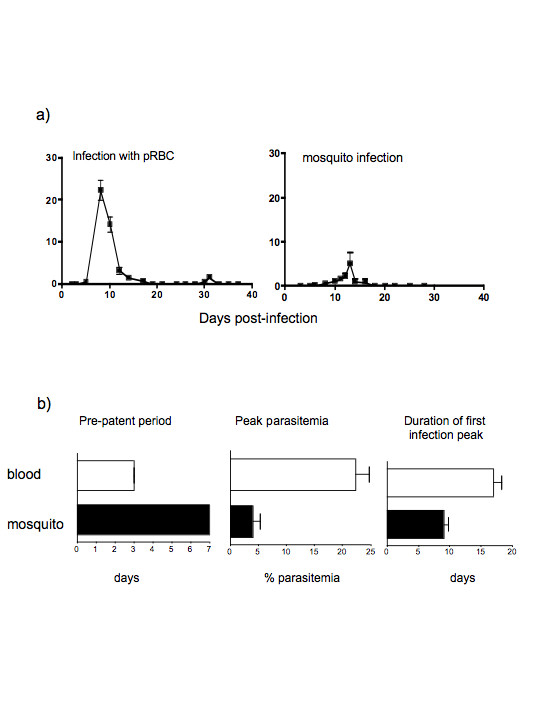
Blood stage infections of *P. chabaudi *(ER) in C57BlC57BL/6 mice initiated by bite of infected mosquitoes and injection of parasitized erythrocytes (pRBCs). a) Course of infection after direct challenge with pRBC, and after infectious mosquito bites as described in the materials and methods (representative experiment of 3 performed). The values shown are the geometric means and standard errors of the parasitaemia from a minimum of five mice. b) Duration of the pre-patent period, peak parasitaemia and duration of infection. The values shown are the means and standard errors of the mean of a minimum of 15 mice.

The course of a blood stage infection with *P. chabaudi *(ER) in C57BL/6 mice was similar to that previously reported for *P. chabaudi *(AS) (Figure 1a) [15]. This clone was chosen for mosquito transmission as gametocytes were readily detectable during a blood stage infection, and it has previously been successfully transmitted in the laboratory via mosquitoes (David Walliker, personal communication,). Here, gametocytes were observed on days 6 and 14 of the primary infection. To establish whether *P. chabaudi *(ER) could be transmitted to the insect vector, female *A. stephensi *mosquitoes were fed on infected mice at these times, as described in materials and methods. Mosquitoes that had ingested blood were incubated at 26°C for 8 to 10 days. Dissection of mosquito stomachs revealed that a higher number of oocysts were present in those mosquitoes, which had fed on mice with a 14 day infection (35% infected mosquitoes at day 6 compared with 72.5% at day 14; 6.2 +/- 1.2 oocysts per infected mosquito at day 6 compared with 13.2+/-2.4 at day 14). Mosquito feeding at day 14 was, therefore, adopted for all further experiments.

The presence of oocysts does not necessarily mean that differentiation to infectious sporozoites would be successful. To determine this, mosquitoes fed on day 14 infected mice and incubated for a further 14 days at 26°C were analysed for the presence of sporozoites in the salivary glands, and for their ability to transmit to new mice in their next blood meal. In three independent experiments, more than 65 % of the mosquitoes dissected were infected, although there was a large variation in the mean sporozoite load per mosquito between the three experiments (290 to 2542 sporozoites per mosquito). Fourteen days after feeding mosquitoes on infected mice, transmission of the parasites from those mosquitoes to uninfected mice was attempted. The courses of parasitaemia in the new mice were determined and compared with infection initiated by direct injection of *P. chabaudi *ER infected RBC (pRBC) (Figure 1).

The courses of infection were variable, with pre-patent periods varying between 5 to 15 days and peaks of parasitaemia of 0.027 to 24% over three experiments. In all experiments, with the exception of a single mouse in one experiment, parasitaemia were reduced to subpatent levels by day 18. Recrudescent infections in two experiments were observed at days 20 to 25 and in one experiment, which was carried on for 40 days, parasites were again reduced to subpatent levels by day 37. The development of parasitaemia in mice infected via mosquito was, in general, similar to that of mice injected with pRBC; parasitaemia was distributed in a single curve with short small recrudescences. However, the initial rate of parasite replication was more rapid in the mice infected directly with pRBC, and infections via mosquitoes showed a later day of patency, fewer days of patent parasitaemia, and lower levels of peak parasitaemia compared with blood-challenged mice (Figure 1b).

The variability in patency in mosquito-transmitted parasites may have reflected the variable number of sporozoites in the salivary glands of individual mosquitoes (from 750 to 2,540 per mosquito). Comparison of the day of patency and day of peak parasitaemia with those obtained after direct injection of known numbers of sporozoites suggested that mice infected via mosquitoes could have received between 100 and 16,000 sporozoites (Table 1)

**Table 1:** Comparison of course of infection of *P. chabaudi *(ER) in mice that were infected with known number of sporozoites with those infected via the bite of infectious mosquitoes.

		Mice	Parasitaemia			
			
			Patency (day)	Peak (day)	Peak (%)	Duration (days)
Numbers of sporozoites injected (i.v.)	100	1	5	13	3.86	15
		2	6	6	0.0114	1
		n ± SEM	5.5 ± 0.1	9.5 ± 3.5	1.9 ± 1.9	8 ± 7.0
	500	1	5	11	3.35	9
		2	5	11	1.4	11
		3	5	11	4.96	15
		n ± SEM	5.0 ± 0.0	11.0 ± 0.3	3.2 ± 1	11.7 ± 1.8
	1000	1	5	11	5.41	15
		2	5	11	3.9	11
		3	5	10	3.9	13
		n ± SEM	5.0 ± 0.0	10.7 ± 0.3	4.4 ± 0.5	13.0 ± 1.2
	16000	1	5	8	7.4	13
Mosquito bite		n ± SEM	6.8 ± 0.6	11.5 ± 1.6	5.5 ± 2.5	9.8 ± 2.0

### The host response to the acute stage of a *P. chabaudi *(ER) infection initiated by mosquito bite

**Figure 2:**
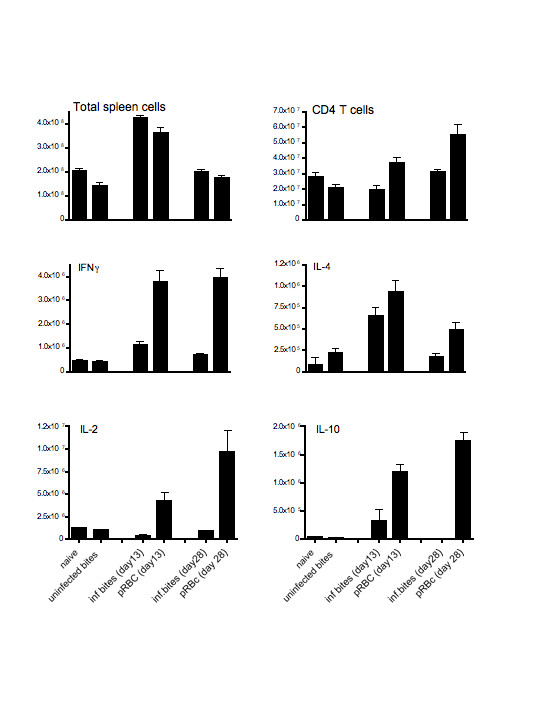
Total numbers of splenic cells (a), CD4^+ ^T cells (b) and CD4^+ ^T cells producing cytokines, IFN-γ (c), IL-4 (d), IL-2 (e) and IL-10 (f) during a primary infection with *P. chabaudi *(ER). Spleen cells taken from mice at days 13 and 28 of infection were counted as described in the materials and methods. CD4^+ ^T cells and cytokines, IFN-γ, IL-2, IL-4 and IL-10, were enumerated by surface and intracellular staining and analysed by FACS. The values shown represent the mean and standard errors of the means from 3 to 5 mice submitted to bites by infected mosquitoes (inf. bites), injected with 10^5 ^parasitized erythrocytes (pRBC), submitted only to uninfected mosquito bites (uninfected bites) and mice not bitten or injected (naive).

The early acute infection of C57BL/6 or BALB/c mice with *P. chabaudi *(AS) is accompanied by splenomegaly, and a large but transient inflammatory response comprising elevated levels of cytokines such as IL-12, TNF-α, IL-6, and IFN-γ [2,6]. Infections with *P. chabaudi *(ER) initiated by injection of pRBC resulted also in increases in spleen size and cellularity similar to those previously reported for the AS clone (Figure 2).

Despite the lower percentage parasitaemia observed in the mosquito-initiated infections compared with direct pRBC infection, at day 13 of infection there were similar increases in numbers of splenic cells (Figure 2a) The total number of CD4^+ ^T cells within the spleen increased above control levels by day 28 in the pRBC-infected mice, but not after infection via the mosquito bites (Figure 2b), suggesting that the impact of direct blood challenge brought about greater CD4^+ ^T cell proliferation.

CD4^+ ^T cells producing cytokines were present in all infected mice at both time points during infection. (Figure 2c–f). In line with the lower numbers of CD4^+ ^T cells, there were significantly less CD4^+ ^T cells expressing IFN-γ, IL-10 and IL-4 in mice infected via mosquito bites than CD4^+ ^T cells in mice injected directly with pRBC (*P *< 0.05, Mann Whitney test). The numbers were however above the levels of normal uninfected mice and mice submitted only to uninfectious bites. In mice infected via mosquito bites, numbers of CD4^+ ^T cells producing IL-2 were also significantly lower (p < 0.05 Mann Whitney test) and were not different from uninfected control values.

### Previous exposure to bites of uninfected mosquitoes affects the course of infection and the CD4^+ ^T cell response during the acute infection

**Figure 3:**
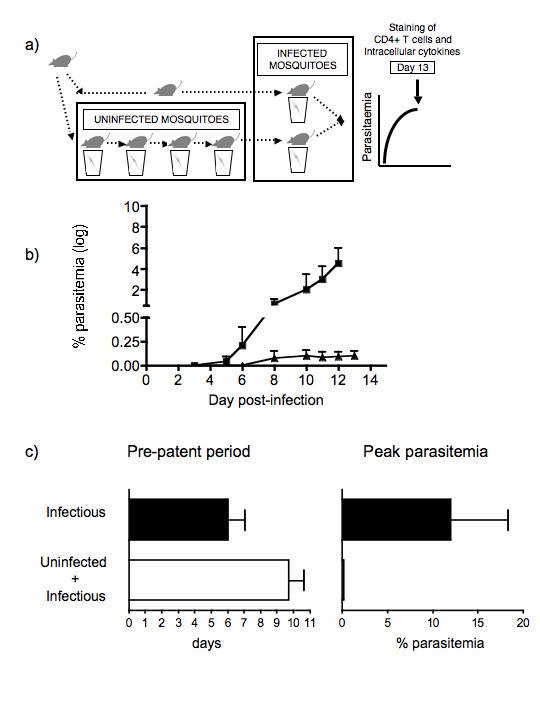
Effects of prior exposure to the bites of uninfected mosquitoes on a blood stage infection of *P. chabaudi *(ER) transmitted by infected mosquitoes. a) Experimental procedure for the infection of mice with *P. chabaudi *(ER) through the bites of infected mosquitoes, after being exposed to four weekly sessions of bites by uninfected mosquitoes. One group of mice was only submitted to infectious mosquito bites. b) Course of infection in mice submitted first to uninfected followed by infected mosquito bites (▲), and infected mosquito bites only (■). The infection was followed for 13 days at which time mice were sacrificed and FACS analysis performed (see Figure 4). c) Length of pre-patent period, and peak parasitaemia of mice submitted to uninfected and infected mosquito bites (□), and infected mosquito bites only (■). The values represent the means and standard errors of the means of at least 3 mice per group.

In nature, the vertebrate host is exposed not only to mosquitoes carrying malaria parasites, but also to the bites of uninfected mosquitoes, commonly of more than one genus and species. Several previous sessions of uninfected *A. stephensi *mosquito bites clearly influenced the patency of parasitaemia, as well as the level and day of peak parasitaemia in mice that were then submitted to bites by mosquitoes infected with *P. chabaudi *(ER). One group of mice was submitted to four weekly sessions of bites by uninfected mosquitoes, the other remained unbitten. One week after the last session of uninfected mosquito bites both groups of mice were exposed to bites by infected mosquitoes (Figure 3a).

After exposure to infected mosquito bites the infection course was monitored for up to 13 days (Figure 3b). Both groups of mice showed patent parasitaemia. However, the day of patency in mice previously submitted to uninfected mosquito bites was three days later and the mice experienced significantly lower peak parasitaemia (p < 0.05, Student's T test), than in those mice submitted only to infectious mosquito bites (Figure 3b–3c).

**Figure 4:**
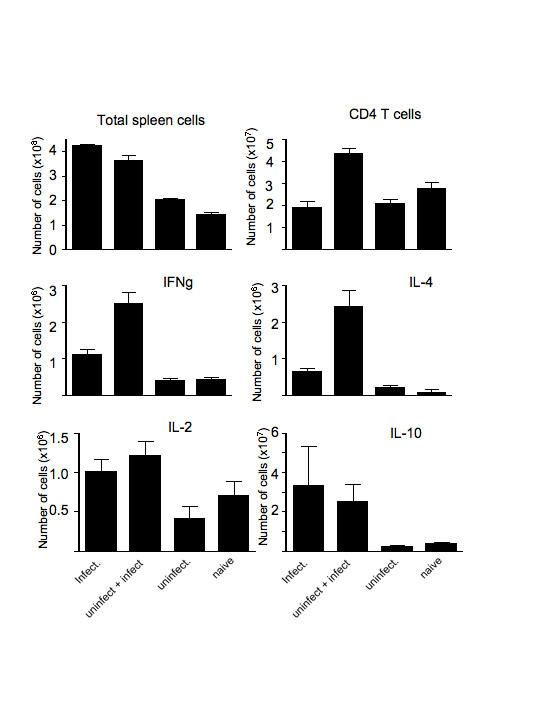
Total numbers of splenic cells (a), CD4^+ ^T cells (b) and CD4^+ ^T cells producing cytokines, IFN-γ, IL-2, IL-4 and IL-10 (c-f respectively) in mice infected with *P. chabaudi *(ER) on day 13 after infection. Mice were submitted to bites by infected mosquitoes (infect), previous bites by uninfected mosquitoes before infected mosquitoes (uninfect. + infect.), only bites by uninfected mosquitoes (uninfect.) and not bitten (naive). The values represent the means and standard errors of the means of 3 mice.

The mice in both groups were sacrificed at day 13 and the cytokine profile of CD4^+ ^T cells examined by flow cytometry. Despite the lower parasitaemia in the mosquito-transmitted infection in mice pre-exposed to uninfected bites, the total number of cells in the spleen increased similarly to those mice infected directly with pRBC (see Figure 2a), but contained a greater proportion of CD4^+ ^T cells (Figure 4a and 4b). The numbers of IL-4- and IFN-γ producing CD4^+ ^T cells were also greater in this group of mice, whereas IL-10 and IL-2 producing cells were similar in both groups of infected mice (Figure 4c–4f).

These results clearly show that bites by uninfected mosquitoes before infectious bites can affect the magnitude of the subsequent blood stage infection and the cytokine response of CD4^+ ^T cells.

## Discussion

Transmission of *P. c. chabaudi *(ER) through the natural route of infection results in an erythrocytic stage infection with a delay in patency, lower peak parasitaemia and shorter duration of an acute infection compared with an infection initiated by direct injection of pRBC. Despite these differences, increases in spleen cellularity, and induction of IFN-γ producing CD4^+ ^T cells, albeit at lower levels, were still observed in the acute infection.

The delay in patency and peak parasitaemia was, in general, consistent with the length of time needed to complete the hepatic cycle, which has been shown to last approximately 52 hours in *P. chabaudi *(AS) [16]. The appearance of parasites in the blood circulation later than two days is likely to have been due to injection of the lower numbers of sporozoites, as the numbers of sporozoites present in infected mosquitoes were very variable.

As direct blood challenge is the accepted route of infection for the study of host responses to blood-stage malaria infections, it is important that this reflects the natural infection as far as possible. Splenomegaly is a characteristic of malaria infections, observed in human infections and rodent models [17-19]. In mice, this is thought to be due in part to the haematopoetic response to the high numbers of RBC lost through infection, and to the trapping of leukocytes in the spleen [20-23]. Despite the reduced numbers of pRBC and the shorter duration of the infection, the increase in the cellularity of the spleen was also observed in mice submitted to infectious mosquito bites. This increase was comparable with that seen in mice injected with pRBC, suggesting that factors other than or, in addition to, parasite load play a role in this process.

Interestingly, increases in numbers of CD4^+ ^T cells did not follow the same pattern and were somewhat reduced in infections initiated by mosquito transmission, perhaps reflecting the reduced PAMP or antigenic stimulus of lower parasitaemia. The lower numbers of CD4^+ ^T cells producing IL-2 would support this possibility. In addition to the smaller increase in CD4^+ ^T cells, more importantly there was a greater reduction in CD4^+ ^T cells producing IFN-γ compared with IL-4 producing cells. This suggests that there may be less of a bias towards Th1 responses in these mice, than is seen in mice infected directly with pRBC.

Sporozoites injected by mosquitoes develop initially in the liver, an organ often described as tolerogenic or immunoprivileged [9]. It is possible that uptake of parasite material by dendritic cells or Kupffer cells occurs in the relative absence of inflammation resulting in immuno-regulation rather than activation. A population of B220+ DC have been identified in liver that may have such immunoregulatory properties [24,25] and Kupffer cells from naïve and sporozoite-infected mice have been shown to produce low or negligible amounts of IL-12p40 [10]. Although antigen-presentation and T cell activation of liver-residing pathogens is likely to take place in lymphoid organs in primary infections, there are increased numbers of Kupffer cells and lymphocytes in the liver during infection, and phagocytic cells of the liver could transport parasite antigens to lymph nodes. This may well influence subsequent presentation to, and activation of CD4^+ ^T cells, impacting on those CD4^+ ^T cell responses to antigens shared between liver and blood stages of *Plasmodium*. A detailed analysis of the cytokine profiles of CD4^+ ^T cells specific for parasite antigens expressed at both liver and blood stages, such as MSP-1 [26], which are activated after mosquito-transmitted infections and blood stage infections would address this. In addition to the lower numbers of IFN-γ^+ ^CD4 ^+ ^T cells, there were also fewer IL-10 ^+ ^CD4 ^+ ^T cells. IL-10 is known to reduce the inflammatory response in this infection, mainly characterized by the production of IFN-γ [15]. These results suggest that the lower parasitaemia stimulates less of an inflammatory response and consequently less of a counter-regulatory response. Since much of the pathogenesis of acute blood stage malaria in this model has been ascribed to pro-inflammatory responses induced in part by IFN-γ from Th1 CD4 ^+ ^T cells [1], it will also be important to determine the extent of the pathological sequelae in mosquito-transmitted infections.

In this study, mosquito bites of uninfected *Anopheles *on their own exert an effect over the development of the parasite subsequently injected into the mouse via infectious mosquitoes. This is reflected in later patency of infection, lower peak parasitaemia, and increases in the number of IFN-γ^+ ^and IL-4^+ ^CD4^+ ^T cells in the blood stage of infection in mice given first non-infectious bites. These results indicate that uninfected mosquito bites can potentiate the immune response elicited by the later infectious mosquito bites. These observations are in line with a previous study showing that there is an increased antibody response in animals bitten by anopheles mosquitoes [27].

These effects may be exerted by components within mosquito saliva injected while blood feeding either by stimulating innate inflammatory responses that enhance T cell activation or by inducing host responses that prevent mosquitoes from remaining at the site of the bite. In line with the study described here, saliva and salivary gland proteins from ticks and sand flies have been shown to interfere with the host immune response taking place during a new parasite infection [28-30]. Hyperimmune serum containing high levels of antibodies against uninfected tick saliva components injected into an animal, also prevented ticks from sticking to the skin and led to the inhibition of tick-borne encephalitis virus replication in them [31]. In the same way, mice previously exposed to bites by uninfected sand flies are significantly protected against *Leishmania *infection [32].

However, this is not a general finding that can be extended to all insect/tick borne infections. In contrast to the observations in this study, salivary gland extracts from *Aedes aegypti *have been shown to cause a reduction in splenic cell proliferation and in the production of Th1 and Th2 cytokines [33]. Tick, sand fly and black fly (*Simulium vittatum*) salivary components also can have suppressive effects on the host immune and inflammatory response [34-38]. It was proposed that this allows the vectors to remain on the host for long periods of time and enhances transmission and establishment of the pathogens, although such reductions have not been observed using Culicinae mosquito *Culex quinquefasciatus *[39].

Saliva from blood sucking arthropod vectors has several components, such as anti-platelet aggregatory, anticoagulatory and anti-vasoconstrictory substances [40,41]. The differences in the effects of bites on the host response may therefore be because the relevant components are not present in all types of insect or tick vectors. This certainly requires further investigation.

In summary, direct blood challenge may not fully reflect the early host responses that take place after mosquito bites, or after repeated bites by uninfected mosquitoes, the normal situation for people living in endemic malaria areas. Therefore, it is important that detailed studies of early host responses should include experiments where transmission by the natural route takes place, especially when testing or comparing antigens as potential vaccine candidates.

## Abbreviations

BSA: bovine serum albumin; IL-2, 4, 10: Interleukin 2, 4 10; MSP-1: Merozoite Surface Protein-1; NK cells: Natural Killer cells; pRBC: parasitized red blood cells;

## Authors' contributions

LF carried out and monitored all infections, participated in experimental design and planning, performed the flow cytometry assays and helped draft manuscript.

ES participated in the flow cytometry assays

GB was responsible for the maintenance of mosquitoes and developing the mosquito transmission studies.

JL conceived the study, participated in its design and drafted the final manuscript.

All authors read and approved the manuscript before submission.
